# Optimizing equine standing sedation: **continuous** infusion of detomidine and butorphanol enhances stability but prolongs ataxia

**DOI:** 10.3389/fvets.2025.1606585

**Published:** 2025-08-21

**Authors:** Julia de Assis Arantes, Isabela Peixoto Rabelo, Lucas Bermudes, Milena Domingues Lacerenza, Rubens Peres Mendes, Rodrigo Romero Corrêa, Diego Iwao Yamada, Carlos Augusto de Araújo Valadão, Renata Gebara Sampaio Dória

**Affiliations:** ^1^Department of Veterinary Medicine, Faculty of Animal Sciences and Food Engineering (FZEA), University of Sao Paulo (USP), Pirassununga, SP, Brazil; ^2^Department of Surgery, School of Veterinary Medicine and Animal Science (FMVZ), University of São Paulo (USP), São Paulo, SP, Brazil; ^3^Department of Veterinary Medicine and Surgery, Faculty of Agricultural and Veterinary Sciences (UNESP), São Paulo State University, Jaboticabal, São Paulo, Brazil

**Keywords:** equine dentistry, sedation protocols, infusion techniques, clinical monitoring, anesthetic management

## Abstract

**Objective:**

This study aimed to compare the sedation quality and cardiorespiratory and behavioral effects of detomidine administered intravenously, either in intermittent boluses or as a continuous rate infusion, with butorphanol in horses pre-medicated with acepromazine for odontoplasty procedures.

**Methods:**

A prospective clinical study was conducted with fifteen adult horses randomly assigned to two groups: Bolus group (BG, *n* = 7) and Infusion group (IG, *n* = 8). Both groups received acepromazine premedication, followed by detomidine administration (bolus or infusion). Butorphanol was administered either as a bolus or continuous infusion during oral cavity evaluation (after detomidine). When sedation was inadequate, an additional bolus of detomidine combined with butorphanol was given. Physiological, sedative, and behavioral parameters were evaluated at multiple time points throughout the procedure. Data were analyzed using statistical models to assess differences between groups and across time points.

**Results:**

Continuous infusion of detomidine resulted in superior sedation quality, greater stability, and a reduced need for rescue sedation (*p* < 0.001) compared to bolus administration. Horses in the BG total detomidine consumption was significantly influenced by procedure duration and the number of readministrations (*p* = 0.004). Despite improved sedation quality in IG, ataxia persisted longer post-procedure. Cardiovascular parameters showed expected α2-agonist effects, with transient reductions in heart rate and stable arterial pressure.

**Conclusions and clinical relevance:**

Continuous infusion of detomidine with butorphanol provided more stable sedation, reducing the need for frequent redosing, but also led to prolonged ataxia. Future studies should explore alternative drug combinations to optimize sedation quality while minimizing ataxia and cardiorespiratory effects.

## 1 Introduction

Oral cavity care is essential for maintaining horses' health, wellbeing, and quality of life, whether they are recreational or athletic animals (1). Oral changes linked to dental pain are considered clinical conditions in these animals (2, 3). To avoid oral discomfort, horses adjust their chewing patterns, which can lead to common occlusal problems such as sharp enamel points, hooks, ramps and excessive transverse ridges (3). The goal of odontoplasty is to correct dental occlusion, preserving dental harmony and preventing dental pain, performance loss, and gastrointestinal issues, such as weight loss or colic caused by inadequate chewing (4, 5).

In the past two decades, advancements in materials and techniques for equine dentistry have improved (3). Consequently, sedation and chemical restraint protocols for standing dental procedures have also evolved to meet this growing demand (6–9). The anesthesiologist must ensure the horse remains in a stable quadrupedal position with minimal or no ataxia, maintaining good sedation: calm, quiet, and unresponsive to oral stimuli—including the absence of chewing and tongue movements when the oral cavity is opened and during procedures (10). In addition, it is essential to ensure that the animal does not experience pain or discomfort, or at least that these are minimized to the greatest extent possible.

Sedation for standing procedures involves medications administered as intermittent boluses or continuous rate infusions (8, 11). In Brazil, among the alpha-2-adrenergic agonists, only xylazine and detomidine are officially licensed for use in horses. Romifidine, medetomidine, and dexmedetomidine are not registered for equine use; dexmedetomidine is licensed only for small animals, and medetomidine has no active registration for any species. Despite these regulatory restrictions, the use of romifidine and dexmedetomidine in horses is supported by several studies conducted in other countries, demonstrating their efficacy and safety for equine sedation (12–14). These may be combined with phenothiazines or opioids and are typically used alongside local or perineural blocks during procedures involving painful stimuli (9, 14). Proper sedation ensures effective dental procedures of varying complexity, providing muscle relaxation and analgesia, reduced consciousness, while minimizing complications (13, 15, 16).

Continuous rate infusions are beneficial for long procedures (lasting over 30 min) due to their ability to provide a steady level of sedation compared to intermittent boluses (6). However, horses undergoing dental surgeries like tooth extractions may require double the sedative dose compared to other standing procedures, as they are exposed to prolonged mouth opening and noxious stimuli (8).

Few studies on anesthesia protocols for clinical dental procedures exist compared to other types of standing surgeries. This study aims to compare the sedation quality and cardiorespiratory and behavioral effects of detomidine administered in intermittent boluses or as a continuous rate infusion with butorphanol in horses pre-medicated with acepromazine for odontoplasty procedures. We hypothesized that continuous infusion of detomidine, associated with butorphanol, would provide more stable sedation than repeated boluses in horses undergoing standing odontoplasty.

## 2 Materials and methods

### 2.1 Animals and housing

The study was approved by the Ethics Committee on Animal Use (CEUA) of the Faculty of Animal Science and Food Engineering (FZEA) at the University of São Paulo (USP), Pirassununga campus, under protocol number 1167131219 (ID001412). The research was conducted at the Equine Clinical and Surgery Sector of the Didactic Clinical Hospital Unit (UDCH) at FZEA/USP, in collaboration with the Teaching and Research Support Center (CAEP) at the Equine Dentistry Center (COE) at FMVZ/USP, in Pirassununga-SP. Fifteen client-owned adult horses (10 geldings and 5 mares) of undefined breed, 6–12 years old and weighing 300–500 kg, referred to the hospital for elective odontoplasty, were enrolled. All animals were deemed healthy after physical examination and routine laboratory testing (complete blood count; urea, creatinine, aspartate aminotransferase, alkaline phosphatase and γ-glutamyl transpeptidase). The horses arrived from their home farms, were fasted for 8 h with water *ad libitum*, and were housed individually in stalls throughout the study. Owners provided written informed consent for their animals to participate.

### 2.2 Study design and blinding

This randomized, prospective clinical trial included horses with no previous dental procedures. Treatment solutions were prepared and coded by a third person not involved in data collection; infusions were administered in an identical manner to all horses to maintain blinding. All dental procedures, oral examinations and behavioral assessments were performed by a single operator, while physiological variables were recorded by a second evaluator. Both remained unaware of treatment allocation for the entire study period. Sample size calculation was based on one of the sedation scale parameters (scored from 1 to 5), considering a minimum difference of 1 point between groups as clinically relevant, with an estimated standard deviation of 0.7. Using a significance level of 5% and a power of 80%, the minimum required number of animals per group was estimated to be seven.

### 2.3 Group formation and sedation procedure

On the day of the experiment, the animals were weighed and brought into a temperature-controlled room (23°C). They were then restrained in stocks, where the cervical region was antiseptically prepared for jugular vein cannulation using a 14G catheter (BD Angiocath 14G, Becton Dickinson, São Paulo, Brazil). Following this, an inspection and palpation of the head structures were performed to assess symmetry and movement of the temporal muscles, facial crests, and rostrocaudal and laterolateral motions through external palpation of the dental arcade. The animals were randomly assigned to two groups: Bolus group (BG, *n* = 7) and Infusion group (IG, *n* = 8). Both groups were pre-medicated with 0.05 mg/kg of acepromazine, administered IV (Acepran 1%, Vetnil, Louveira-SP, Brazil). Thirty minutes later, the horses in the BG group received an IV bolus dose of 20 mcg/kg of detomidine Dormiun^®^ V—Agener União, São José do Rio Preto, Brazil, followed by a continuous infusion 1L/h of 0.9% NaCl (KabiPac, Fresenius Kabi Brasil Ltda, Jaguariúna, Brazil), while horses in the IG group received the same bolus dose of detomidine, followed by a continuous infusion of 20 mcg/kg/h of detomidine. The infusion was prepared by diluting detomidine in 1L of 0.9% NaCl and administered using a peristaltic infusion pump (ST1000, Samtronic, Bragança Paulista, Brazil). Ten minutes after the bolus administration, a mouth speculum was positioned for oral cavity evaluation. An oral endoscope was used to visualize and document any alterations, and odontoplasty was subsequently performed. During this phase, all animals received an IV bolus of 7 mcg/kg of butorphanol (Butorfin^®^ 1%—Vetnil, Louveira-SP, Brazil). In the IG, butorphanol was added to the continuous infusion along with detomidine at a rate of 7 mcg/kg/h. Sedation was considered insufficient whenever any of the following variables—sedation quality, tongue activity, chewing, or head movement—received a score < 3. In these cases, the oral speculum was briefly closed, and a rescue dose of detomidine (7 μg kg^−^1) plus butorphanol (7 μg kg^−^1) was administered intravenously. The speculum remained closed for 5 min to allow for full sedative effect before resuming the procedure.

### 2.4 Dental procedures and time points

To identify dental alterations, a thorough oral examination was performed by Evaluator 1 using a mouth speculum, a dental mirror, and a 70-cm-long rigid endoscope with a 90° optical angle (GDI oroscope, Brazil). Each dental arcade was individually assessed for changes in occlusal wear, the presence of infundibular or peripheral caries, diastemata, pulp exposures, and fractures. The findings were recorded in specific dental charts along with photographic documentation. Subsequently, all horses underwent odontoplasty to achieve occlusal equilibration. The following time points were used for the collection of data: before the detomidine bolus administration (T0), and 5, 15, 30, 45, 60, 75, 90, and 105 min after its administration (T5, T15, T30, T45, T60, T75, T90, T105, respectively), as well as 15 and 30 minutes after the completion of the odontoplasty (15P and 30P, respectively).

### 2.5 Physiological, sedative, and behavioral assessments

For each animal, physiological parameters (mucous membrane color, capillary refill time, heart rate, respiratory rate, non-invasive blood pressure, intestinal motility, and rectal temperature) were evaluated as described in Table 1. Sedation quality and behavioral responses were assessed based on ataxia, sedation score, tongue movement, chewing and head movements, oral response to water stimulation, head height, and overall behavior during the procedure, as detailed in Table 2. Additionally, procedure time, the number of readministrations, and the total administered dose of detomidine and butorphanol were recorded.

**Table 1:** Physiological parameters: assessment methods and evaluation time points.

**Parameter**	**Method of evaluation**
Mucosas^a^	Digital exposure and classified into scores: 1 (pale), 2 (pink), 3 (congested)
Capillary refill time^a^ (TPC)	Pressure on the gum near the upper incisors for 5 s, done in duplicate
Heart rate^a^ (HR)	Measured by auscultation in the left intercostal space (3rd or 4th) for 1 min
Respiratory rate^a^ (RR)	Indirectly by counting rib cage excursions for 1 min
Non-invasive blood pressure^a^ (NIBP)	Non-invasive oscillometric measurement of systolic (SAP), diastolic (DAP), and mean (MAP) arterial pressure using a multiparameter monitor (MEC 1200Vet, Mindray), in triplicate. The cuff width corresponded to 40% of the tail circumference, and measurements were corrected for hydrostatic effects by adding 0.736 mmHg per centimeter of vertical distance between cuff and right atrium. Mean values were used for analysis.
Rectal temperature^a^ (RT)	Measured with a mercury thermometer for 3 min
Intestinal motility^a^	Auscultation of four abdominal quadrants for 3 min, noting signs of tympany, hypomotility, or hypermotility

^a^Timepoints: T0, T5, T15, T30, T45, T60, T75, T90, T105, 15P, 30P.

**Table 2:** Sedation quality and behavioral responses: scoring criteria and evaluation time points.

**Parameter**	**Score**	**Observation**
Head height^a^	(%)	Measured with a tape measure from the lower lip to the ground, 1 min after head release from the dental headgear. Values were expressed as a percentage relative to T0 (100%)
Ataxia^a^	1	Leaning on stocks/crossed pelvic limbs/flexed thoracic limbs
	2	Oscillating, sometimes leaning on stocks
	3	Oscillating with constant support changes
	4	Normal limb support with oscillation
	5	Absent
Sedation quality^b^	1	No sedation
	2	Mild sedation with strong response to intervention
	3	Moderate sedation with partial response to stimulus
	4	Good sedation with minimal response
	5	Deep sedation, no response
Tongue movement^b^	1	Intense tongue movement
	2	Moderate tongue movement
	3	No movement, reactive to touch
	4	No movement, reactive to manual traction
	5	No movement, no response to touch
Chewing and head movement^b^	1	Unable to keep mouth speculum^d^
	2	Intense chewing and abrupt head movement
	3	Chewing with repeated head movements
	4	Chewing without repetitive/abrupt head movements
	5	No chewing or repetitive movements
Response to oral water stimulation^b^	1	Tongue movement, chewing, and head movement
	2	Tongue movement and chewing
	3	Moderate tongue movement
	4	Subtle tongue movement
	5	No reaction
Overall behavior during the procedure^c^	1	Impossible: procedure abandoned due to poor sedation
	2	Poor: completed with difficulty due to strong interference
	3	Fair: completed with moderate interference
	4	Good: minimal interference
	5	Excellent: no interference

^a^Timepoints: T0, T5, T30, T60, T90, T105, 15P, 30P.

^b^Timepoints: T15, T30, T45, T60, T75, T90, T105.

^c^Timepoints: Single evaluation after the end of the procedure.

^d^Excessive movement and reactivity preventing safe maintenance of the mouth speculum.

### 2.6 Statistics

Statistical analyses were performed using GraphPad Prism (version 7.00) and SPSS (version 25), considering a significance level of *p* ≤ 0.05. Data normality was assessed using the Shapiro-Wilk test. Parametric data were analyzed using repeated-measures ANOVA (RM-ANOVA), followed by Dunnett's test for comparisons over time and *t*-tests for comparisons between groups. For non-parametric data, the Friedman test was used for comparisons over time, followed by Dunn's test, while the Mann–Whitney test was applied for comparisons between groups. To assess the relationship between the number of detomidine readministrations and the variables procedure duration and experimental group, a Poisson regression model was employed. Additionally, multiple linear regression analysis was performed to evaluate the association between the total administered dose of detomidine, the number of readministrations, and procedure duration.

## 3 Results

### 3.1 Physiological parameters

**Figure 1:**
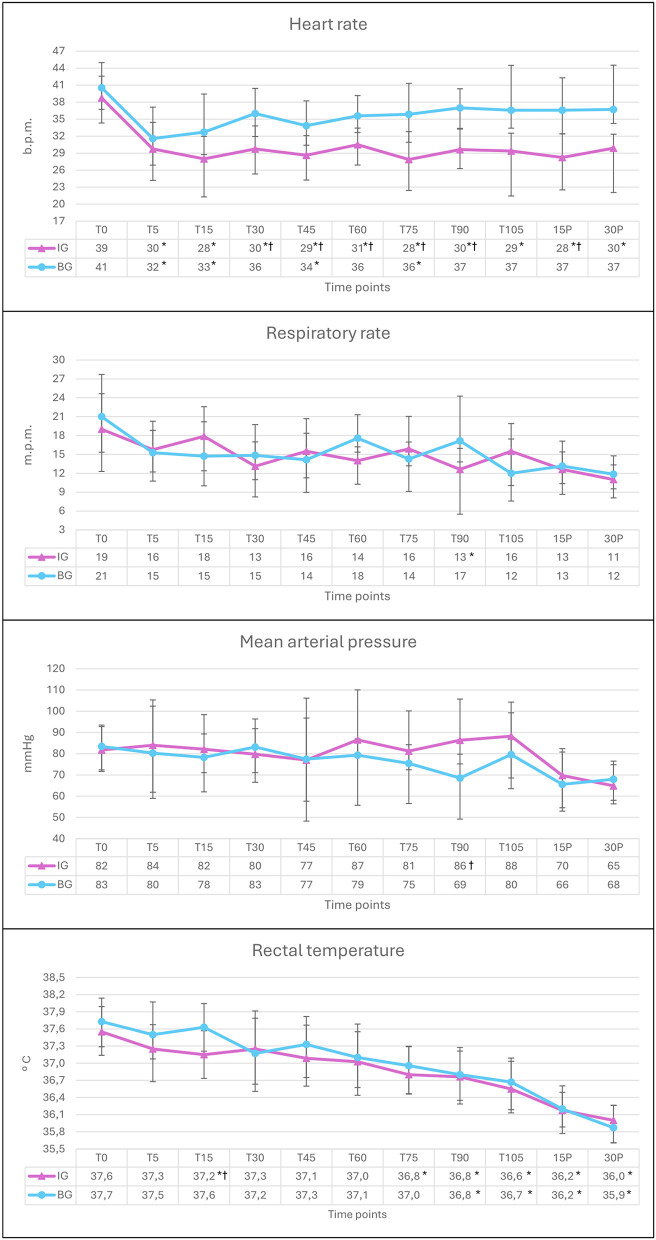
Graphical representation of the means and standard deviation of heart rate, respiratory rate, mean arterial pressure and rectal temperature in horses pre-medicated with acepromazine and treated with detomidine via continuous infusion (IG, *n* = 8) or bolus (BG, *n* = 7) at baseline (T0), 5, 15, 30, 45, 60, 75, 90, and 105 min after detomidine administration, as well as 15 and 30 min after odontoplasty (15P and 30P). *Different from T0, in the same group, by Dunnett's test (*p* < 0.05).^†^Different from BG, by *T*-test (*p* < 0.05).

Graphical variations of physiological parameters are illustrated in Figure 1. Heart rate (HR) showed differences over time in both groups. In IG, variations (0.0005 < *p* < 0.0262) were observed between baseline and all subsequent timepoints. In the BG, HR varied (0.012 < *p* < 0.0365) between T0 and T5, T15, T45, and T75. Between groups, differences (0.0013 < *p* < 0.0266) were found at T30, T45, T60, T75, T90, 15P. Respiratory rate (RR) showed variation over time only in the IG, between T0 and T90 (*p* = 0.0373), with no differences between groups. Rectal temperature (RT) varied over time in both groups. In IG, differences (0.0001 < *p* < 0.0353) were observed between T0 and T15, T75, T90, T105, 15P, and 30P. In BG, variation (0.0001 < *p* < 0.0475) was found between T0 and T90, T105, 15P, and 30P. Between groups, a difference was observed at T15 (*p* = 0.0451). Systolic arterial pressure (SAP) and mean arterial pressure (MAP) showed differences between groups at T90 (SAP: *p* = 0.0240; MAP: *p* = 0.0482). Diastolic arterial pressure (DAP) showed no significant differences over time or between groups.

### 3.2 Sedative and behavioral assessments

**Figure 2:**
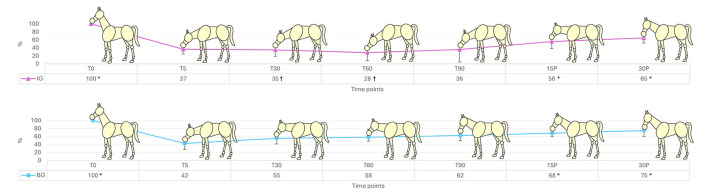
Graphical representation of head height (%) in horses pre-medicated with acepromazine and treated with detomidine via continuous infusion (IG, *n* = 8) or bolus (BG, *n* = 7) at baseline (T0), 5, 30, 60, and 90 min after detomidine administration, as well as 15 and 30 min after odontoplasty (15P and 30P). *Different from the lowest level of heigt (T60 in IG and T5 in BG), by Dunnett's test (*p* < 0.05).^†^Different from BG, by *T*-test (*p* < 0.05).

As shown in Figure 2, head height showed the greatest reduction at T60 in the IG (28 ± 9.5%) and at T5 in the BG (42.6 ± 12.1%). Within-group comparisons revealed differences over time relative to T60 in IG and T5 in BG at T0 (IG and BG: *p* < 0.0001), 15P (IG: *p* = 0.0007; BG: *p* = 0.0115), and 30P (IG: *p* = 0.0010; BG: *p* = 0.0041). Between-group differences were observed at T30 (*p* = 0.0328) and T60 (*p* = 0.0078). Ataxia showed differences over time compared to baseline in IG (0.001 < *p* < 0.014) at T30, T45, T60, T75, T90, and T105, and in BG (0.005 < *p* < 0.025) at T5, T15, T30, T60, and T75, with no differences between groups. Median scores for other parameters are presented in Table 3. Tongue movement differed between groups at T90 (IG = 5 vs. BG = 3, *p* = 0.0140). No significant differences were found between time points or groups for sedation quality, chewing movements, or response to oral water stimulation. Overall behavior during the procedure differed between groups (*p* = 0.0242), with better scores in IG (Median: 5—Excellent) compared to BG (Median: 4—Good). As shown in Table 4, procedure time did not differ between groups. However, the number of readministrations and the total dose of detomidine were significantly different. No differences were observed in the total dose of butorphanol.

**Table 3:** Median (inferior limit; superior limit) in horses pre-medicated with acepromazine and treated with detomidine via continuous infusion (IG, *n* = 8) or bolus (BG, *n* = 7) 15, 30, 45, 60, 75, and 90 min after detomidine administration.

**Parameter**	**Group**	**T15**	**T30**	**T45**	**T60**	**T75**	**T90**
Sedation quality (1–5)	IG	4 (3; 5)	4 (4; 5)	3 (3; 5)	4 (3; 5)	5 (3; 5)	4 (4; 5)
	BG	5 (4; 5)	4 (3; 5)	4 (2; 5)	5 (4; 5)	4 (3; 5)	4 (3; 5)
Tongue movement (1–5)	IG	4 (3; 5)	4 (4; 5)	4 (3; 5)	4 (3; 5)	5 (3; 5)	5 (4; 5)^†^
	BG	4 (3; 5)	4 (2; 5)	4 (2; 5)	5 (3; 5)	4 (3; 5)	3 (2; 5)
Chewing and head movement (1–5)	IG	5 (4; 5)	5 (4; 5)	5 (3; 5)	4 (3; 5)	5 (4; 5)	5 (4; 5)
	BG	4 (3; 5)	4 (3; 5)	4 (3; 5)	5 (3; 5)	4 (3; 5)	4 (3; 5)
Response to oral water stimulation (1–5)	IG	5 (2; 5)	5 (4; 5)	5 (3; 5)	5 (3; 5)	5 (3; 5)	5 (4; 5)
	BG	4 (2; 5)	4 (2; 5)	4 (2; 5)	4 (2; 5)	4 (2; 5)	4 (2; 5)

^†^Different from BG, by Mann–Whitney test (*p* < 0.05).

**Table 4:** Means ± standard deviation of procedure time, number of readministrations, and total doses of detomidine and butorphanol in horses pre-medicated with acepromazine and treated with detomidine via continuous infusion (IG, *n* = 8) or bolus (BG, *n* = 7).

**Parameter (unit)**	**Group**	**X ± SD**	***P*-value**
Procedure time (min)	IG	127.5 ± 16.9	0.3044
	BG	140.1 ± 26.5	
Readministrations (n°)	IG	1.6 ± 1.2^†^	0.0182
	BG	4.3 ± 1.9	
Total dose detomidine (mcg/kg)	IG	75.1 ± 13.0^†^	0.0090
	BG	50.0 ± 13.2	
Total dose of butorphanol (mcg/kg)	IG	29.6 ± 11.1	0.2646
	BG	37.0 ± 13.2	

^†^Different from BG, by Mann–Whitney test (*p* < 0.05).

The Poisson regression model revealed that the number of detomidine readministrations was significantly influenced by both the experimental group and procedure duration. Horses in the Bolus Group (BG) required significantly more rescue sedation than those in the Infusion Group (IG) (B = 18.997, *p* < 0.001). Additionally, for every 1-h increase in procedure duration, the number of readministrations increased significantly (B = 11.141, *p* = 0.005). However, the interaction between group and procedure duration indicated that this effect was less pronounced in the BG (B = −4.924, *p* = 0.006), suggesting that while both groups required more rescue as surgery time increased, this increase was more gradual in the BG.

The multiple linear regression analysis demonstrated that both procedure duration and the number of readministrations were significantly associated with the total administered dose of detomidine. The first model, which included only procedure duration, explained 82.6% of the variability in total detomidine dose (*R*^2^ = 0.826). When readministrations were added as a predictor, the model improved significantly (*R*^2^ = 0.916, *p* = 0.004), showing that both factors contribute to detomidine consumption. In the final model, procedure duration remained the strongest predictor (B = 18.365, *p* = 0.002), but the number of readministrations also had a significant effect (B = 2.900, *p* = 0.004).

## 4 Discussion

### 4.1 Physiological parameters

Reflex bradycardia resulting from increased peripheral vascular resistance is a well-documented effect of α2-adrenergic receptor agonists (7, 9, 12). A reduction in heart rate and an increase in arterial pressure have been reported in multiple clinical studies involving α2-agonists in horses (8, 9, 12). In the present study, the decrease in HR was more pronounced in the IG, where values remained significantly lower than baseline throughout all time points, including 30 min after the infusion was discontinued. In contrast, in the BG, HR reduction was observed only up to T75, suggesting a shorter duration of the cardiovascular effects when detomidine was administered as a bolus. Despite the observed decrease in HR, values remained within acceptable physiological limits for the species, indicating that the reduction was not clinically concerning (17).

Interestingly, despite the reduction in heart rate (HR), mean arterial pressure (MAP) remained stable over time in both groups, suggesting effective cardiovascular compensation. α2-adrenoceptor agonists are known to induce peripheral vasoconstriction, leading to an increase in systemic vascular resistance (SVR), which in turn triggers reflex bradycardia via baroreceptor activation. According to the hemodynamic relationship “MAP = (HR × SV) × SVR,” the rise in SVR likely played a key role in maintaining MAP despite the observed bradycardia. However, since cardiac output was not directly measured, it is not possible to conclude whether perfusion remained entirely unaffected. Further studies evaluating tissue oxygenation and cardiac output would be necessary to confirm whether systemic perfusion was preserved under these sedation protocols. Although overall stability was observed, the decrease in MAP at T90 in the BG suggests that bolus administration, along with repeated dosing, may be associated with more abrupt hemodynamic fluctuations.

Rectal temperature gradually decreased over time in both groups, likely due to the combined effects of phenothiazines and α2-agonists, both known to influence thermoregulation. While α2-agonists affect the central nervous system and heat production (18), phenothiazines induce vasodilation and suppress thermoregulatory mechanisms (19). Although hypothermia is generally more severe under general anesthesia due to hypothalamic depression and vasodilation (20), in this study, the animals remained sedated in a quadrupedal position, which may have helped preserve some thermoregulatory responses and reduced the clinical impact of the temperature drop. However, this effect should still be considered in prolonged procedures to avoid potential complications.

### 4.2 Sedative and behavioral assessments

Both protocols were safe and allowed the completion of odontoplasty. However, continuous infusion offered advantages in terms of sedation stability and reduced need for rescue sedation. Overall behavior during the procedure was significantly better in the IG, and head lowering, a key indicator of sedation depth with α2-agonists (14, 21), was more pronounced in this group. Additionally, Poisson regression confirmed that horses in the BG required significantly more readministrations than those in the IG (B = 18.997, *p* < 0.001), reinforcing the idea that continuous infusion provides more sustained sedation, minimizing fluctuations in drug effect. Despite the relatively frequent re-administration of sedation in the BG (mean 4.3 top-ups per horse), each rescue maintained a fixed dose of detomidine and butorphanol (7 μg kg^−^1 each), preserving the pharmacodynamic synergism of the combination. The cumulative butorphanol dose remained below 30 μg kg^−^1 h^−^1 in all cases—a range considered clinically acceptable and not associated with increased ataxia in similar standing procedures (22).

The total detomidine dose was higher in the IG, which was expected given the continuous infusion rate per hour was 20 mcg/kg, exceeding the bolus dose used for readministrations in both groups (7 mcg/kg). In contrast, the butorphanol doses were identical (7 mcg/kg in bolus or per hour). Since the BG required more frequent readministrations, the total butorphanol dose in both groups was similar. Multiple linear regression analysis further demonstrated that both procedure duration and the number of readministrations were significantly associated with detomidine consumption. While procedure duration was the strongest predictor (B = 18.365, *p* = 0.002), the number of readministrations also played a significant role (B = 2.900, *p* = 0.004), confirming that both factors influenced total drug use.

Neither protocol maintained continuous, stable sedation without the need for redosing, particularly in the BG, leading to repeated interruptions for drug administration. A study that also evaluated the continuous infusion of detomidine and butorphanol in horses reported that 7 out of 8 animals required rescue doses (9); however, the number of readministrations per animal was not specified. In the present study, 6 out of 8 animals needed supplementation, with a mean of 1.6 ± 1.2 rescues per animal. Despite the lower butorphanol dose used in our protocol (7 mcg/kg/h vs. 30 mcg/kg/h), the need for readministration was not higher, which may be attributed to the greater detomidine dose both in the initial bolus (20 mcg/kg vs. 10 mcg/kg) and in the continuous infusion (20 mcg/kg/h vs. 10 mcg/kg/h). Additionally, premedication with acepromazine could have contributed to a prolonged sedative effect through a synergistic action with alpha-2 agonists, potentially reducing the need for additional interventions.

Both protocols also induced significant ataxia, which persisted longer in the IG (up to 15 min post-procedure). This effect is well-documented for α2-agonists, particularly detomidine (23). In contrast, a study by Müller et al. (13) reported that the combination of α2-agonists with butorphanol or ketamine did not increase ataxia, whereas the association with midazolam resulted in significantly greater ataxia in sedated horses. Although a previous study reported less ataxia with buprenorphine and levomethadone compared to butorphanol during dental extractions (24), no differences were found in sedation quality or physiological parameters. Given that odontoplasty involves milder nociceptive stimuli and deep sedation from detomidine, we prioritized clinical feasibility. Future studies should evaluate μ-agonist opioids in more painful procedures to assess potential benefits in analgesia and ataxia control, and investigate whether increasing the butorphanol dose while reducing the α2-agonist dose, or combining these agents with other drugs, could enhance sedation quality and minimize the need for supplemental boluses.

### 4.3 Limitations

This study has several limitations that should be acknowledged. Oral procedures in horses are inherently challenging due to the requirement for unnatural positioning and the potential for variable behavioral responses. Furthermore, despite including only horses without prior odontoplasty, variations in the severity of oral lesions may have influenced individual sensitivity and responses during manipulation, potentially impacting the evaluations. Another limitation was the absence of plasma concentration measurements for detomidine and butorphanol, which precluded direct correlation between pharmacokinetic and pharmacodynamic effects. Monitoring plasma drug levels could have provided a more precise understanding of drug metabolism, clearance, and their relationship with sedation quality and duration. Additionally, the lack of a control group receiving detomidine alone limits the ability to isolate the effects of the α_2_-agonist, as well as to evaluate the potential enhancing effect of butorphanol on sedation. Future research should explore alternative drug combinations, such as α2-agonists combined with benzodiazepines, ketamine, or other opioids—including full μ-agonists and partial μ-agonists—to determine whether these protocols provide more stable sedation with fewer adverse effects.

## 5 Conclusions

Both sedation protocols allowed the completion of odontoplasty safely and effectively. However, continuous infusion of detomidine with butorphanol provided more stable and prolonged sedation, reducing the need for repeated drug administration. Horses in the bolus group required significantly more readministrations, resulting in greater fluctuations in sedation depth. Despite these advantages, continuous infusion was associated with prolonged ataxia, which may present a challenge for post-procedure recovery. Cardiovascular effects were consistent with α2-agonist administration, with transient heart rate reduction and stable arterial pressure. Future studies should evaluate alternative drug combinations that enhance sedation quality while mitigating ataxia and other side effects, ensuring optimal conditions for equine standing dental procedures.

## Data Availability

The raw data supporting the conclusions of this article will be made available by the authors, without undue reservation.
